# Stress estimation by serum cortisol release during rapid maxillary expansion in mice: validation of an experimental model

**DOI:** 10.1590/2177-6709.30.4.e252518.oar

**Published:** 2026-01-09

**Authors:** Rodrigo RODRIGUES, Jose Alejandro GUERRERO, Anna Alice ANABUKI, Jôice Dias CORRÊA, Raquel Souto SILVA, Vivien Thiemy SAKAI, Tarcília Aparecida da SILVA, Heloisa Sousa GOMES, Soraia MACARI

**Affiliations:** 1 Federal University of Alfenas, Dentistry Course (Alfenas/MG, Brazil).; 2 Universidad del Rosario, Dentistry Course, Department of Mechanical Engineering (Bogotá, Colombia).; 3 Federal University of Goiás, Dentistry Course (Goiânia/GO, Brazil).; 4 Pontifical Catholic University of Minas Gerais, Dentistry Course, Department of Dentistry (Belo Horizonte/MG, Brazil).; 5 Federal University of Minas Gerais, Dentistry Course, Department of Pathology and Oral Surgery (Belo Horizonte/MG, Brazil).; 6 Federal University of Minas Gerais, Dentistry Course, Department of Restorative Dentistry (Belo Horizonte/MG, Brazil).

**Keywords:** Biomarkers, Orthodontics, Cortisol, Palatal Expansion Technique, Physiological stress, Biomarcadores, Ortodontia, Cortisol, Técnica de Expansão Palatina, Estresse fisiológico

## Abstract

**Introduction:**

Rapid maxillary expansion (RME) is an orthopedic procedure used to correct maxillary transverse deficiencies by applying forces through specialized appliances. This intervention induces both dental and skeletal changes, which may potentially elicit physiological stress responses.

**Objective:**

To validate a model for estimating stress by measuring plasma cortisol levels in mice subjected to rapid RME.

**Methods:**

5-6-week-old male mice (C57BL6/J) were submitted to RME performed by an opening loop distractor at the mid palatal suture calibrated as follows (n = 5 per group): no force (control); 0.28 Newtons (N), 0.42N and 0.56N for periods of 7 and 14 days. Histomorphometry analyses were performed to analyze RME effect and serum samples were collected to measure cortisol by enzyme-linked immunosorbent assay (ELISA).

**Results:**

The forces applied at two different time points resulted in a successful RME pattern with the opening of the mid-palatal suture compared to the control group (P<0.05). The 0.42N force at 7 days resulted in a significant (P<0.05) increase in cortisol (pg/ml) compared to the control and 0.28N at 7 days (d) groups; the cortisol level in the 0.42N 7d group was statistically reduced when compared to the 0.42N 14d group. There was no statistically significant difference between the other groups.

**Conclusion:**

The results suggest an increase in the stress response during the first days after the application of RME force with a force of 0.42N, with subsequent body adaptation. The force parameter of 0.42N at days proved to be a valid model for analyzing RME in mice.

## RESUMO

**Introdução:**

Expansão rápida da maxila (ERM) é um procedimento ortopédico usado para corrigir deficiências transversais maxilares pela aplicação de forças por meio de aparelhos especializados. Essa intervenção induz alterações dentárias e esqueléticas, que podem potencialmente provocar respostas fisiológicas de estresse.

**Objetivo:**

Validar um modelo para estimar o estresse medindo os níveis plasmáticos de cortisol em camundongos submetidos à ERM rápida.

**Métodos:**

Camundongos machos de 5 a 6 semanas de idade (C57BL6/J) foram submetidos à ERM realizada por um distrator de alça de abertura na sutura palatina mediana calibrada da seguinte forma (n = 5 por grupo): sem força (controle); 0,28 Newtons (N), 0,42N e 0,56N por períodos de 7 e 14 dias. Análises de histomorfometria foram realizadas para analisar o efeito da ERM e amostras de soro foram coletadas para medir o cortisol por ensaio imunoenzimático (ELISA).

**Resultados:**

As forças aplicadas em dois momentos diferentes resultaram em um padrão de ERM bem-sucedido caracterizado pela abertura da sutura palatina mediana em comparação ao grupo controle (P<0,05). A força de 0,42N em 7 dias resultou em um aumento significativo (P<0,05) no cortisol (pg/ml) em comparação aos grupos controle e 0,28N em 7 dias (d); o nível de cortisol no grupo 0,42N 7d foi estatisticamente maior quando comparado ao grupo 0,42N 14d. Não houve diferença estatisticamente significativa entre os outros grupos.

**Conclusão:**

Os resultados sugerem um aumento na resposta ao estresse durante os primeiros dias após a aplicação da força de ERM com uma força de 0,42N, com subsequente adaptação corporal.

## INTRODUCTION

Rapid maxillary expansion (RME) is an effective orthodontic treatment for correcting maxillary width deficiencies by opening the mid-palatal suture to correct malocclusions such as posterior crossbites.^1^ ^,^ ^2^ The application of forces is required to expand the mid-palatal suture and produce adequate orthopedic repositioning with less tooth movement.^1^ ^,^ ^3^

Although there are no negative side effects on general health concerning RME,^2^ the exerted forces can change the state of other facial structures producing side effects on the temporomandibular joint, median palatine suture, root resorption^4^ and, muscle activity.^2^ ^,^ ^5^ This procedure produces zones of tension and compression in the sutural regions and teeth, respectively, causing pain, for example.^6^ ^-^ ^8^

A painful experience may lead to physiological stress^9^ and the development of internal stress,^8^ altering biological events in rodents^10^ ^,^ ^11^ and humans.^12^ When a stressful stimulus occurs, cortisol is the main glucocorticoid hormone released into the bloodstream.^13^ ^-^ ^15^ Some studies have shown an increase in serum cortisol in mice after stressful situations, such as restraint^16^ ^,^ ^17^ swimming and unpredictable stress.^17^

Although there is little evidence regarding cortisol release during RME, a previous study demonstrated higher cortisol levels during this procedure at different times in children.^18^ One of the major concerns of patients and professionals regarding orthodontic treatment is the discomfort caused by the physical effects of the appliances.^18^ Thus, the ability to identify how mechanical forces influence physiological stress may provide information to improve strategies for RME in clinical practice. Therefore, this study aimed to analyze and validate a maxillary rapid maxillary expansion (RME) model in mice by measuring serum cortisol levels, in order to estimate the stress associated with the RME disjunction procedure.

## MATERIALS AND METHODS

### ANIMALS

This study followed the animal pre-clinical study (ARRIVE) guidelines. Eight-week-old male mice (C57Bl/6) were obtained from the Central Animal Facility of the Federal University of Minas Gerais (Brazil). All animals were treated under the ethical standards for animal experiments, as defined by the Institutional Ethics Committee (Approval Protocol No. 152/2016). The animals were acclimated to experimental conditions such as living in plastic cages, food, and water ad libitum and maintained under a 12-h light/dark cycle.

### EXPERIMENTAL INDUCTION OF RME

The mice were anesthetized intraperitoneally (100 mg/kg of ketamine plus 10 mg/kg of xylazine) and placed in dorsal decubitus, allowing complete visualization of the intraoral structures. A previously activated 0.014-inch opening loop (GAC International Inc) was made and bonded to the occlusal surface of the upper first and second molars on both sides using a light-cured resin (Transbond, Unitek/3M, Monrovia, CA, USA). The magnitude of the force was calibrated by a strain gauge (Shimpo Instruments, Itasca, IL, USA) to exert forces of 0.28 Newtons (N), 0.42N, and 0.56N as described previously.^19^ The springs were calibrated to ensure consistent force delivery.^19^ There was no reactivation during the experimental period. Animals with non-activated passive springs were used as control (control group). After the springs were installed, the animals were fed a soft diet. The mice were euthanized by decapitation under anesthetics at the following times: 7 and 14 days after placement of the orthodontic appliance, and the jawbone and blood were collected. Five mice were used for each experimental group and time point. During the experimental period, the animal’s weight was recorded and there was no significant weight loss.

### HISTOMORPHOMETRY AND RME MEASUREMENT

The maxillary bones were dissected and immediately immersed in buffered formalin solution, pH 7.2, for 48 hours for fixation. The pieces were then washed in 14% EDTA solution (pH 7.4) for 20 days with daily solution changes. Afterward, the pieces were washed for 4 hours in running water. To obtain standardized longitudinal sections, a cut in the coronal direction close to the first upper molars was made with a razor. The portion of the maxillae containing the incisors was cut and discarded. Only the fragments containing the three upper molars on both sides were used as samples. After the preparation described, routine histological processing was performed with dehydration in increasing series of 70%, 80%, 90%, and absolute alcohol, with the fragments remaining immersed for 30 minutes in each alcohol. Subsequently, the specimens were clarified in 3 xylol baths (Mono 2000 Tissue Processor, Lupe Indústria e Comércio Ltda, São Carlos, Brazil). At the end of the histological processing, the samples were embedded in paraffin, with the palatal surface facing the microtomy plane. The paraffin blocks were cut at 5 µm thickness using a rotary microtome (Jung, Histocut 820, Mussioch, Germany). The selected slides of the maxilla were then stained with Masson’s Trichrome according to the manufacturer’s instructions.

**Figure 1:**
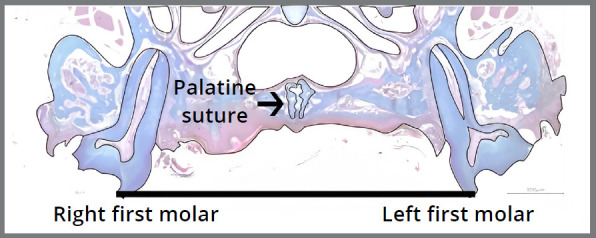
Histological section and graphic image (black line) demonstrating the site of analysis of rapid maxillary expansion (RME) in millimeters (mm). The RME was measured using a line parallel to the occlusal plane between the points of the apex of the palatine cusp of the upper left first molar to the apex of the palatine cusp of the upper right first molar in histological sections at 10x magnification.

The slides with histological sections were photographed under an optical microscope connected to a digital camera (PowerShot A620, Canon, Tokyo, Honshu, Japan) at 10x magnification. The images obtained were analyzed using the software Image J (National Institutes of Health, USA). The RME was measured in millimeters (mm) using a line parallel to the occlusal plane between the points of the apex of the palatal cusp of the upper left first molar to the apex of the palatal cusp of the upper right first molar (Fig. 1). For each animal, the RME was defined as the mean of the total area between the cusp tips evaluated on the palatal cusps of the first molars of the two hemiarches. The measurements were performed blindly by a researcher unaware of the treatments to which the animals were subjected. The well-being of the animals after RME was monitored by tracking changes in their body weight.

### ASSESSMENT OF SERUM CORTISOL LEVELS

Blood samples were placed in 1.5 ml tubes and centrifuged at 4,000 rpm for 10 minutes at 4°C to obtain serum and stored in a freezer at -80°C until analyzed. Blood cortisol levels were assessed by double-binding enzyme-linked immunosorbent assay (ELISA) according to the manufacturer’s protocol (Enzo Life Sciences Kit - New York, NY, USA). The results were expressed as picograms of cortisol per ml of serum (pg/ml).

### STATISTICAL ANALYSIS

Data were expressed as mean ± standard deviation (SD), with P < 0.05 considered statistically significant using Two-way ANOVA followed by Bonferroni’s multiple comparisons test.

## RESULTS

**Figure 2:**
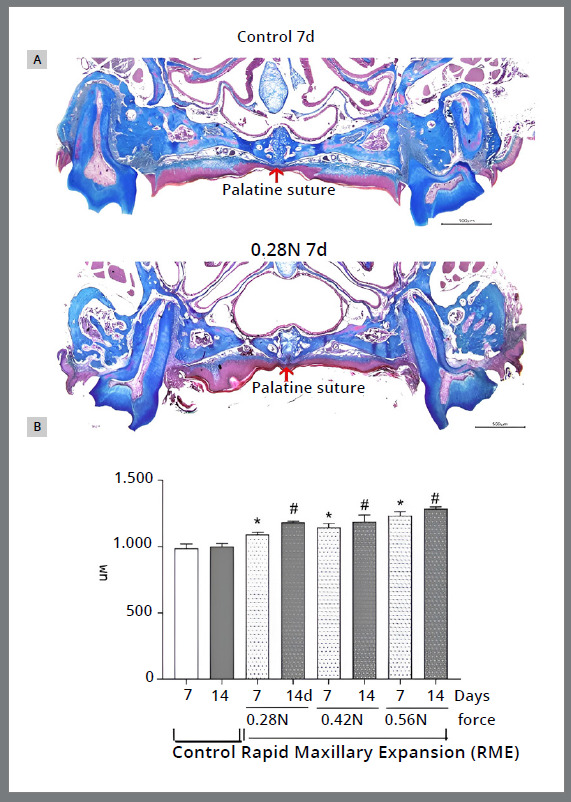
A) Illustrative image demonstrating rapid maxillary expansion measured between the upper right first molar and the upper left first molar of mice in the control and 0.28 Newtons (N) groups over 7 days. B) Results of RME disjunction in millimeters (mm) (n = 5 mice per experimental group). *P < 0.05 different from 7d control. ^#^P < 0.05 different from 14d control.10x magnification. Two-way ANOVA followed by Bonferroni’s multiple comparisons test.

Palatal suture expansion was successful in all animals. All three forces applied at two different time points resulted in a similar pattern with an increase in the RME distance compared to the control group (Fig. 2 and Table 1). The spring used for rapid maxillary expansion interfered with the feeding behavior of animals in the experimental groups, leading to a statistically significant reduction in their body weight (Table 2).

**Table 1:** Rapid maxillary expansion (RME) distance in millimeters (mm) and standard deviation (S.D.). Palatal suture expansion was successful in all experimental groups (n = 5 mice per experimental group). A similar letter means no statistical difference. Different letter means. P < 0.005. Two-way ANOVA followed by Bonferroni’s multiple comparisons test.

Groups	Control		0,28N		0,42N		0,56N	
	7 days	14º day	7 days	14º day	7 days	14º day	7 days	14º day
Mean (mm)	9844^a^	9996^a^	10924^b^	11819^b^	11430^b^	11862^b^	12350^b^	12846^b^
S.D. (±)	769.4	447	305.6	174.7	667.5	1172	612.9	319,1

**Table 2:** Weight of the animals after rapid maxillary expansion (RME) in grams (g) and standard deviation (S.D.). Palatal suture expansion was successful in all experimental groups (n = 5 mice per experimental group). A similar letter means no statistical difference. Different letter means P < 0.005. Two-way ANOVA followed by Bonferroni’s multiple comparisons test.

Groups	Control		0,28N		0,42N		0,56N	
	7 days	14º day	7 days	14º day	7 days	14º day	7 days	14º day
Mean (mm)	20.85^a^	22.66^b^	17.64^c^	19.33^c^	17.48^c^	19.26^d^	17.03^c^	17.07^c^
S.D. (±)	1.816	1.047	0.4248	2.643	0.38	2.016	0.4461	0.4472

**Figure 3:**
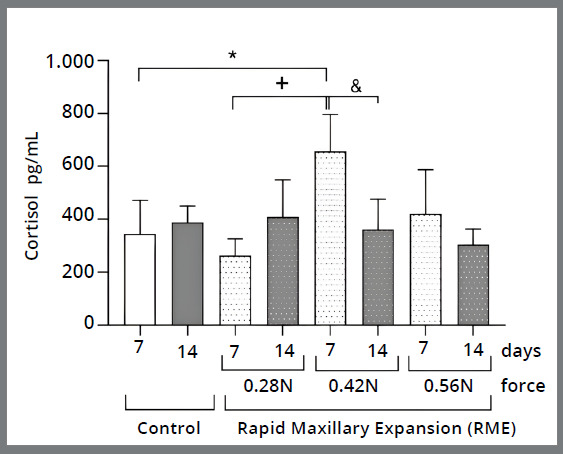
Results of cortisol levels in the control and experimental (with RME) groups with forces of 0.28 Newtons (N), 42N, and 0.56N at 7 and 14 days. *P < 0.05 different from 7d control. ^+^P < 0.05 different from 7d 0.28N. ^&^P < 0.05 different from 14d 0.42N (n = 5 mice per experimental group). Two-way ANOVA followed by Bonferroni’s multiple comparisons test.

There was a significant increase in cortisol levels in the 0.42N group (657.0 pg/ml ± 138.9), with a statistically significant difference when compared to the control group (345.8 pg/ml ± 125.4) over the 7 days (7d). The cortisol level in the 0.42N 7d group was significantly higher than in the 0.28N 7d (264.0 pg/ml ± 61.75) and 0.42N 14 d (361.4 pg/ml ± 114.6) groups. There was no difference when comparing the cortisol levels of the other groups (Fig. 3).

## DISCUSSION

The results of the RME protocol showed that the applied force of 0.42N caused an increase in cortisol levels on day 7 and, decreased levels on day 14, during suture expansion in mice, suggesting increased stress response during the first days after RME. Furthermore, sutural expansion occurred successfully in all animals with similar RME patterns in all three forces applied at both time points tested.

Correct cementation and activation of the device are essential for successful mechanics.^2^ Several RME techniques in rats^4^ ^,^ ^20^ and mice^19^ ^,^ ^21^ have been proposed in the literature. In this study, a 0.014-inch open spring previously activated was used and then bonded to the occlusal surface of the upper first and second molars on both sides using a light-cured resin according to Guerrero et al.^19^ Animals with non-activated loops were used as controls. The use of 0.014-inch stainless steel orthodontic wire as an open loop to promote expansion proved to be efficient, corroborating previous studies.^19^ ^,^ ^21^ ^-^ ^23^ The RME had a successful impact in the mid-palatal suture opening measured by histomorphometry analysis and using the palatal cusp of the upper first molars as a reference to measure the transverse maxillary expansion, as already proven through computed tomography-micturition analysis in the literature.^19^ ^,^ ^21^

The orthodontic procedure of rapid maxillary expansion creates zones of tension and compression due to ischemia and inflammation in the periodontal ligament^24^ resulting in a painful experience that is related to a stress response.^11^ ^,^ ^18^ ^,^ ^25^ The pain reported by patients may not be directly related to the magnitude of the force applied, but rather to the psychological conditions of the patients.^25^ ^,^ ^26^ Physical signs of pain in mice can be observed through changes in behavior and posture, as well as facial expressions that reflect discomfort.^27^ In our study, the animals exhibited changes in feeding and social behaviors, though without significant weight loss. These changes recurred within two days and were alleviated by a soft diet.

Mechanical stress and inflammatory mediators can produce oxidative stress.^19^ The study by Gecgelen and collaborators,^18^ using saliva samples from individuals before and after RME treatment, showed higher cortisol levels on the first day of device installation compared to other days, with a statistically significant difference. The cause, according to the authors, may be associated with the stress and anxiety that the beginning of use of the device can cause in patients.^18^ Gecgelen et al.^18^ also observed that in the following days after RME, the cortisol level was maintained, which may be associated with adaptation to the expander. This study showed that cortisol levels were higher in the first 7 days after the application of forces of 0.42N and decreased after 14 days, corroborating and resembling the results of Gecgelen and collaborators in children.^18^

Previous studies have found that cortisol levels were similar to controls when patients adapted to the expander device.^18^ This could explain the decrease in cortisol levels after 14 days compared to the 7 days presented in this study. These findings are consistent with other studies that suggest a reduction in anxiety levels by the end of treatment.^6^ ^,^ ^18^

Cortisol is the main glucocorticoid involved in the regulation of stress responses in rodents, being a responder to acute stress.^17^ Some studies have used cortisol levels as an assessment to identify stress in mice in different situations such as restraint, swimming, and unpredictable stress.^9^ ^,^ ^17^ ^,^ ^28^ ^,^ ^29^ In these studies, the method used to measure cortisol includes radioimmunoassay,^17^ and also ELISA,^30^ as was done in the present study. In future studies, the concomitant analysis of salivary and plasma cortisol may be relevant to understanding the dynamics of stress during RME in mice and a better comprehension of the physiological response to stress and pain. Hardy and collaborators^31^ explain that among the hormones produced by the adrenal gland, cortisol has the most important impact on bone. Although the synthesis of adrenal cortisol and androgens is part of normal physiology, there are also effects of the action of these hormones in pathological states.^32^ Given the above, further investigations are needed to assess whether the increased cortisol level during RME would be similar to physiological or pathological changes.

The limitation is the use of an animal model, since the literature lacks randomized clinical studies evaluating cortisol levels in RME. However, the study presents ease of reproducibility of the model, reliability of results, and corroborates other findings^18^, which can provide important information to improve RME strategies in studies and clinical practice.

## CONCLUSION

The maxillary RME model in mice, analyzed through cortisol levels, proved to be reliable and reproducible. An increased response to stress was also observed during the first days after the application of RME force with subsequent body adaptation.
